# Adaptive Visual Re-Weighting in Children’s Postural Control

**DOI:** 10.1371/journal.pone.0082215

**Published:** 2013-12-04

**Authors:** Paula F. Polastri, José A. Barela

**Affiliations:** 1 Faculty of Sciences, UNESP - University Estadual Paulista, Bauru, Department of Physical Education, Laboratory of Information, Vision, and Action, Bauru, São Paulo, Brazil; 2 Institute of Physical Activity and Sport Sciences, Cruzeiro do Sul University, São Paulo, São Paulo, Brazil; 3 Institute of Biosciences, UNESP - Univ Estadual Paulista, Rio Claro, Department of Physical Education, Rio Claro, São Paulo, Brazil; Bielefeld University, Germany

## Abstract

This study investigated how children’s postural control adapts to changes in the visual environment and whether they use previous experience to adjust postural responses to following expositions. Four-, eight-, and twelve-year-old children (10 in each group) and 10 young adults stood upright inside of a moving room during eight trials each lasting one-minute. In the first trial, the room was stationary. In the following seven trials, the room oscillated at 0.2 Hz, amplitude of 0.5 cm, with the exception of the fifth trial, in which the room oscillated with amplitude of 3.2 cm. Body sway responses of young adults and older children down-weighted more to the increased visual stimulus amplitude when compared to younger children. In addition, four- and eight-year-old children quickly up-weighted body responses to visual stimulus in the subsequent two trials after the high amplitude trial. Sway variability decreased with age and was greatest during the high-amplitude trial. These results indicate that four year olds have already developed the adaptive capability to quickly down-weight visual influences. However, the increased gain values and residual variability observed for the younger children suggest that they have not fully calibrated their adaptive response to that of the young adults tested. Moreover, younger children do not carry over their previous experience from the sensorial environment to adapt to future changes.

## Introduction

 Living in an environment with sensory changes requires us to continuously modify the coupling strength between the available sensory stimuli and our body orientation. Such a mechanism not only requires one to identify coherent relationships between the acquired inputs and the action performed but also to skillfully modify this relationship as the environmental demands change. Despite the importance of such a behavior, little is known about the underlying sensorimotor re-weighting mechanisms. The lack of information is even more egregious when trying to deduce this behavior with regard to development.

 Sensory information manipulation induces coherent postural responses even in infants [1,2] and such use of sensory information continues to improve throughout the first decade of life [3-5]. However, infants and children show more difficulties in properly resolving situations with conflicting sensory information and they might even fall as a result [6–8]. In a pioneer study, Lee and Aronson [6] demonstrated that infants and younger children were substantially influenced by the surrounding visual information while standing in a “moving room”, in which postural responses to the room’s movement resulted in a large number of falls and staggers, suggesting poor stability and a predominance of the use of visual information to maintain the upright stance at an early age [6,8,9].

It has been suggested that children below seven years of age are unable to properly integrate sensory inputs coming from multiple sensory systems, i.e., visual, somatosensory, and vestibular, with prevalence for using visual information [6,7,10-14]. On the other hand, several studies have not suggested any sensory input dominance on postural control functioning [3,5,15-17]. Moreover, recent results indicated that developmental changes in sensorimotor integration occur even after the first decade of life [5,16,18]. 

Integration of inputs coming from multiple sensory systems implies that the central nervous system (CNS) accurately detects and re-weights sensory inputs which are providing the most reliable and useful information in a specific condition as the environmental conditions change. Use of inputs coming from multiple sources implies an elegant system that obtains precise information about the body in the environment which then uses this obtained information to estimate the body’s position and velocity which is crucial for maintaining or achieving desired postural orientation and equilibrium [19]. Moreover, this system demands a complex mechanism in which the importance of the sensory input is constantly adjusted to adapt to new conditions in which sensory input importance continuously varies.

Although Forssberg and Nashner [7] first recognized the importance of sensory integration for postural development, only recently has sensorimotor re-weighting been systematically examined in adult and elderly postural control [20-25]. Based upon these studies, nonlinear models of postural responses to sudden amplitude changes of the visual surrounding have contributed to the understanding of some aspects of the dynamics of sensorimotor re-weighting [26-29]. Experimental results have also indicated that when the visual stimulus amplitude is abruptly increased or decreased, then the body sway to the driving stimulus amplitude is decreased or increased, respectively [25]. Similarly, when one is standing and a visual flow is unexpectedly created, correspondent body sway is induced until the CNS decreases visual cues use in favor of cues coming from other sensory sources (i.e., vestibular and somatosensory) and regains body stabilization [30]. Therefore, stimulus amplitude-dependent body responses have been interpreted as sensorimotor re-weighting. Thus, hypothetically the CNS attributes less weight to visual input when the amplitude is increased or unexpectedly created in order to functionally avoid any threat to postural stability [25,26].

Although some studies have provided some evidence that children are able to adapt to changes in sensory inputs [3,5,31], only recently, have a few studies investigated sensorimotor re-weighting capabilities in children’s postural control [17,32], thereby elucidating some of the mechanisms underlying the development of postural control over the years. For instance, intra-modality re-weighting to visual input amplitude has been shown in children as young as 4 years old [17,32]. In this case, intra-modal re-weighting was interpreted as the changes (increases/decreases) in the coupling between visual information and body sway due to changes (decreases/increases) in the visual stimulus amplitude.

Besides showing adaptive responses in children’s postural control due to changes in the visual stimulus amplitude (low-to-high and high-to-low amplitude conditions), Rinaldi, Polastri and Barela [32] have also shown that children’s responses to sensory changes are uncalibrated, unlike adult responses. Moreover, adult-like postural control responses, during such changes, are not observed until after the first decade of life, with larger magnitude responses observed in younger children as compared to 12-year-old children and young adults. Younger children also exhibited these responses after a larger visual stimulus amplitude unlike older children and adults. Such a behavior might indicate that children are unable to use previous experiences in sensory changes to the following exposition.

A system that is both improperly calibrate and that does not carry over previously to the following experiences might be compromised more and, during conflicting sensory situations, result in falls [c.f., 6] and discomfort and disruption of postural orientation and equilibrium [7]. Certainly, continuous modification of the coupling between sensory stimuli and body sway is a signature feature of flexible and stable postural orientation that seems to be acquired through experience. Unfortunately, it remains unclear how experience in a continuously changing environment can modify the dynamics of sensorimotor re-weighting in children’s postural control and amount of time this modification requires. In a previous study from our group, this aspect was only briefly evaluated because changes in visual stimuli occurred within a trial and any inspection of adaptation among exposition was impossible. Thus, the aim of this study was to investigate the adaptation of children’s postural control during changes in the surrounding visual environment and whether a children’s postural control uses previous experience to adjust its response during subsequent expositions.

## Materials and Methods

### Subjects

Thirty healthy children approximately 4-years-old (mean age = 3.7 years, ± 2.6 months, five females, five males), 8-years-old (mean age = 8.1 years, ± 3.3 months, five females and five males) or 12-years-old (mean age = 12.1 years, ± 5.7 months, five females and five males) and ten healthy young adults between the ages of 20 and 27 years old (mean age = 22.0 years, ± 2.4 years, six females and four males) participated in this study. The study was approved by the Ethics Committee of Bioscience Institute, UNESP – Univ Estadual Paulista - Campus of Rio Claro. All participants were treated according to the ethical standards of the National Committee of Ethics in Research (CONEP), Brazilian Government. All children’s parents or guardians were informed about the experimental procedures for this study and provided a completed written consent form approved by the local Institutional Review Board. Adults also provided written consent. 

### Procedures

 After a brief acclimatization period in the laboratory environment, each participant stood in an upright position inside of a “moving room”. This room was composed of three white walls covered by vertical black stripes (22 cm – width) and a white roof mounted on four wheels (2.1 x 2.1 x 2.1 m – width, height and length, respectively). The wheels were placed on rails allowing the entire structure to continuously move backwards and forwards independent of the floor. A servomechanism comprised of a controller (Compumotor – APEX 6151), a servo motor (Compumotor – N0992 GRONMDN) and an electrical cylinder (Compumotor – APEX620-MO-NC) produced the room’s movement that was controlled by custom software (Compumotor - Motion Architect for Windows). 

 An infrared emitter (Optotrak – Digital Northern, Inc) placed on the participant’s back (near the eighth thoracic vertebra) and another placed on the room’s frontal wall were used to obtain information about the participant’s body sway and the room’s position in three directions (anterior-posterior, medio-lateral and vertical), respectively. Information from each of these emitters was sampled at 100 Hz. 

 The participants were asked to stand as still as possible, 1 m from the front wall of the room, looking at an infantile picture placed at eye level for 8 trials, with each trial lasting 60 seconds. In the first trial, the room remained stationary. In the following three trials, the room was oscillated with an amplitude of 0.5 cm and a peak velocity of 0.6 cm/s (low amplitude/velocity condition). In the fifth trial, the room was oscillated with an amplitude of 3.2 cm and a peak velocity of 4.1cm/s (high amplitude/velocity condition). Finally, in the last three trials, the room was oscillated with the low amplitude/velocity condition parameters (an amplitude of 0.5 cm and a peak velocity of 0.6 cm/s). A 60-second resting period was provided after the third and the sixth trials to prevent muscle fatigue or inattentive behavior during the test. 

Headphones were provided to the participants to reduce any auditory noise from the laboratory environment. Six of the youngest children refused to wear these headphones, however no difference was found in their results compared to the other youngest children in the same group. A digital video camera (Panasonic – WV-CL350) was placed behind the room’s front wall and this camera allowed observation of the participants’ test to determine if they were looking at the picture.

In addition, previous knowledge about the room’s movement might affect postural responses to the visual stimulus [33,34] and, therefore, the participants were naïve to the room’s motion. After each trial, participants were asked if they had noticed anything different during the trial. Only two 12-year old children and six young adults reported that the walls were oscillating, after they were exposed to the high amplitude/velocity condition. Nine children (three of the 8-year-olds and six of the 12-year-olds) and four young adults reported increased self-motion or discomfort during the high amplitude/velocity condition, but they were completely unaware of any room movement. 

### Data Analysis

Since the visual stimulus manipulation was in the anterior-posterior (AP) direction, analyses were performed to recorded data in this direction. For each participant in each trial a frequency-response function (FRF) was computed from body sway and the visual stimulus. More specifically, the FRF was calculated by dividing the Fourier transforms of body oscillation by the Fourier transforms of the visual stimulus, generating a complex-valued function (transfer function). 

From the transfer function values, gain, phase and sway variability (position and velocity variability) were calculated for each trial and then averaged across groups to verify the effect of the visual stimulus on the body sway at the driving frequency (0.2 Hz). Gain was computed as the absolute value of the transfer function and indicated the coupling strength between visual stimulus and body sway. A gain value of 1 indicated that the spectrum amplitude of body sway was equal to the spectrum amplitude of the room’s movement. Phase was computed as the argument of the transfer function, converted into degrees, and indicated the temporal relationship between visual stimulus and body sway. Positive phase values indicate that body sway led the room’s movement and negative phase values indicate that body sway was behind to the room’s movement. 

Position and velocity variability of body sway was computed as the standard deviation of the sway trajectory [35] after the component of the body sway due the stimulus frequency was removed (residual trajectories). Position and velocity variability values indicate body sway amplitude and velocity (sway variability), respectively, at frequencies other than the 0.2 Hz frequency with higher values indicating higher variability.

In addition, mean sway amplitude was computed for all trials by calculating the standard deviation of the body sway time series after the average of the body sway position was subtracted from the data points within each trial and then averaged across groups. Mean sway amplitude indicates the overall body sway throughout each trial with lower values indicating better maintenance of an upright stance. 

### Statistical Analysis

 The F-statistic was tested from the absolute value and argument of the average of the FRF across all groups to verify if the FRF values (real and imaginary parts) were different from zero, which would indicate that the phase values have not been misled by low gain values causing the gain results to be overestimated [35]. Univariate normal distribution for FRF values was assumed. This analysis revealed that FRF values were different from zero (p < 0.05) among all groups and trials. 

From these results, gain, phase, position and velocity variability measurements were tested as dependent variables for four repeated measures ANOVAs (4 groups x 7 trials) to verify whether changes of the visual stimulus (amplitude and velocity) had affected body sway responses and whether this effect was different among groups. Another repeated measures ANOVA was also performed to examine differences among and within-group conditions on mean sway amplitude at AP direction. Finally, a one-way ANOVA was performed in order to examine differences among groups for the mean sway amplitude at AP and medio-lateral (ML) directions during the stationary trial. Post-hoc analyses with Tukey’s correction were performed when necessary. The α-level for these analyses was 0.05. All analyses were performed using SPSS software (SPSS version 10.0). All results are presented as means ± SD.

## Results

 Participant’s body oscillation was induced by visual manipulation, with sway observed at the same frequency of the driving signal across all trials. 

### Mean sway amplitude

**Figure 1:**
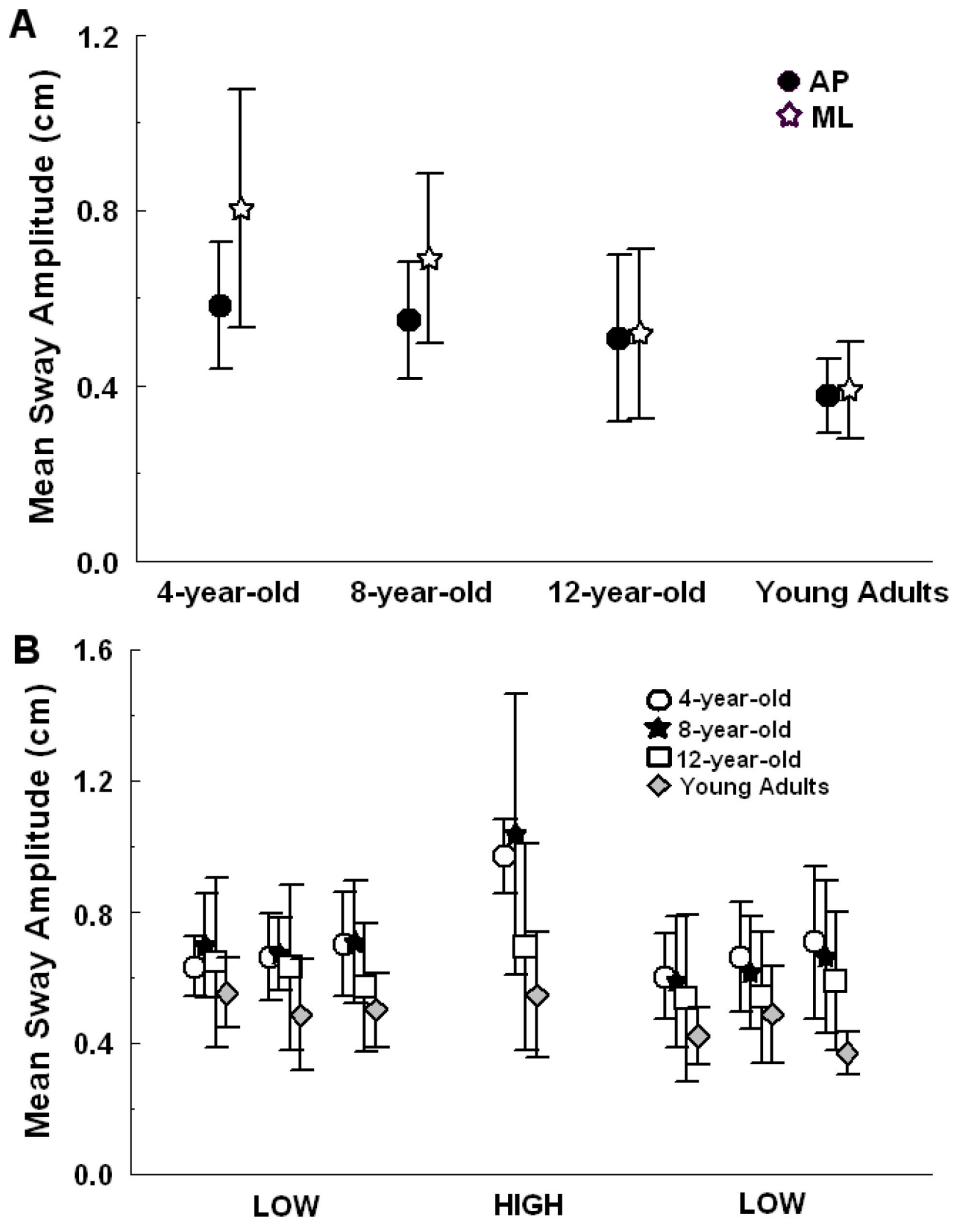
Mean sway amplitude. Mean sway amplitude values of each age group (4-, 8- and 12-year-old and young adults) at room’s stationary trial at anterior-posterior (AP) and medio-lateral (ML) directions (A) and lower and higher-amplitude trials at anterior-posterior (AP) direction (B). Values are mean ± SD.

Figure 1 depicts the mean sway amplitude for each age group in the room’s stationary trial (Figure 1a) and in all trials when the room was oscillated (Figure 1b). Age-related differences in body sway magnitude were observed in the room’s stationary trial. ANOVA indicated mean sway amplitude group effect for both ML direction, F(3,37) = 7.32, p < 0.001, and AP direction, F(3,37) = 3.29, p < 0.04. Post-hoc tests indicated, in the ML direction, larger mean sway amplitude for the 4-year-old group when compared to the 12-year-old group (p <0.02) and young adults (p < 0.001) and a larger mean sway amplitude for 8-year-old group compared to young adults (p<0.02). In the AP direction, only the 4-year-old group presented larger mean sway amplitude when compared to the young adults (p < 0.03).

In the trials in which the room was oscillated, ANOVA revealed trial, F(6,216) = 14.122, p < 0.001, and group effects, F(3,36) = 5.925, p < 0.002, and a group and trial interaction, F(18,216) = 2.082, p < 0.01. Post-hoc tests indicated that children’s body oscillations were larger in all trials when compared to young adults. Only the 4-year-old and 8-year-old groups exhibited increased body oscillation magnitude in the high amplitude/velocity trial when compared to any low amplitude/velocity trial (p < 0.04). Finally, there were no differences in the body sway magnitude between the 12-year-old group and young adults under the high amplitude/velocity trial condition (p > 0.05). 

### Gain and Phase

**Figure 2:**
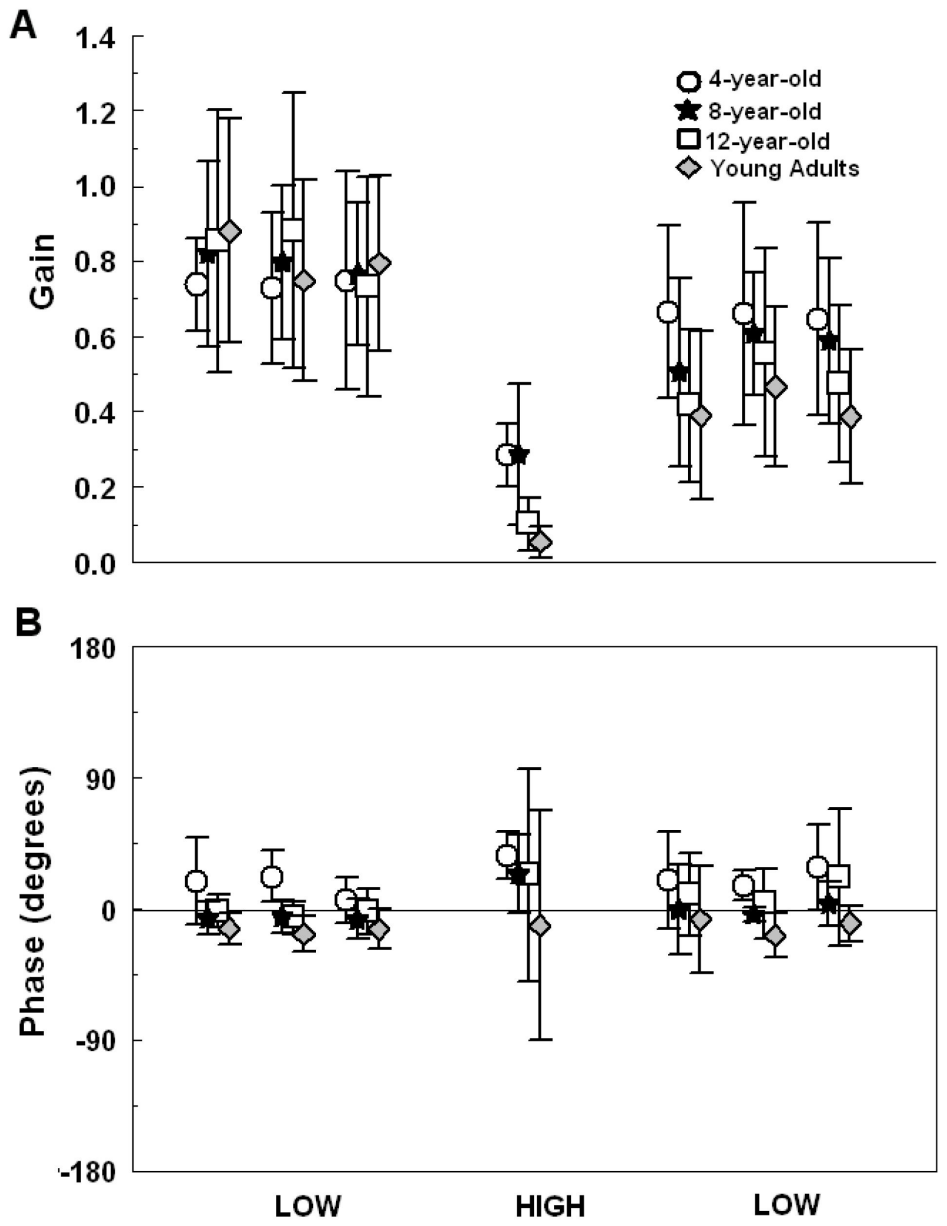
Gain and phase between visual stimulus and body sway. Gain (A) and phase values (B) for each age group (4-, 8- and 12-year-old and young adults) across all trials. Values are mean ± SD.

Figure 2a depicts gain values for each age group across all trials. In general, participant’s body oscillation was strongly influenced by the visual stimulus except in the high amplitude/velocity trial condition. Also, group and trial interaction was found in the body sway responses in the trials following the amplitude/velocity change in the visual stimulus. ANOVA revealed gain trial effect, F(6,216) = 104.501, p < 0.001, and a group and trial interaction, F(18,216) = 4.001, p < 0.001, but no group effect, F(3,36) = 1.835, p > 0.05. Post-hoc analyses showed that body sway amplitude decreased at the higher-amplitude condition when compared to the lower-amplitude trials, indicating reduced coupling to the visual stimulus (p < 0.002). However, body responses from the 12-year-old group and young adults down-weighted more to the stimulus when compared to the 4-year-old and 8-year-old groups (p < 0.008). Moreover, the 12-year-old group and young adults showed lower gain values in the trials following the high amplitude/velocity trial when compared to the previous trials (p < 0.05) (low condition – Figure 2a). A similar effect was not observed for the 4-year-old group and the 8-year-old group (p > 0.05). The 4-year-old group quickly up-weighted body responses to the visual stimulus in the trials following the high amplitude/velocity trial, with gain values similar to those observed previously during the high amplitude/velocity trial. The eight-year-old group also up-weighted body responses to the visual stimulus after the high amplitude/velocity trial but showed gain values similar to those observed previously to the high amplitude/velocity trial only after the sixth trial. 

Figure 2b depicts phase values for each age group across all trials. ANOVA revealed phase group, F(3,36) = 17.779, p < 0.001, and trial effect, F(6,216) = 3.013, p < 0.01, but no group and trial interaction, F(6,216) = 0.395, p > 0.05. Post-hoc tests revealed that the temporal relationship of the 4-year-old group was different when compared to the 8- and 12-year-old groups (p < 0.03), and also that young adults were different when compared to all three children groups (p < 0.04). Body responses of the youngest groups slightly led the visual stimulus as indicated by the positive phase values found. On the other hand, the 12-year-old group demonstrated phase values close to zero, indicating body responses temporally close to the visual stimulus. Finally, young adults demonstrated negative phase values, indicating body responses temporally delayed to the visual stimulus. Post-hoc tests did not revealed differences in phase values among trials within each age group. 

### Position and Velocity Variability

**Figure 3:**
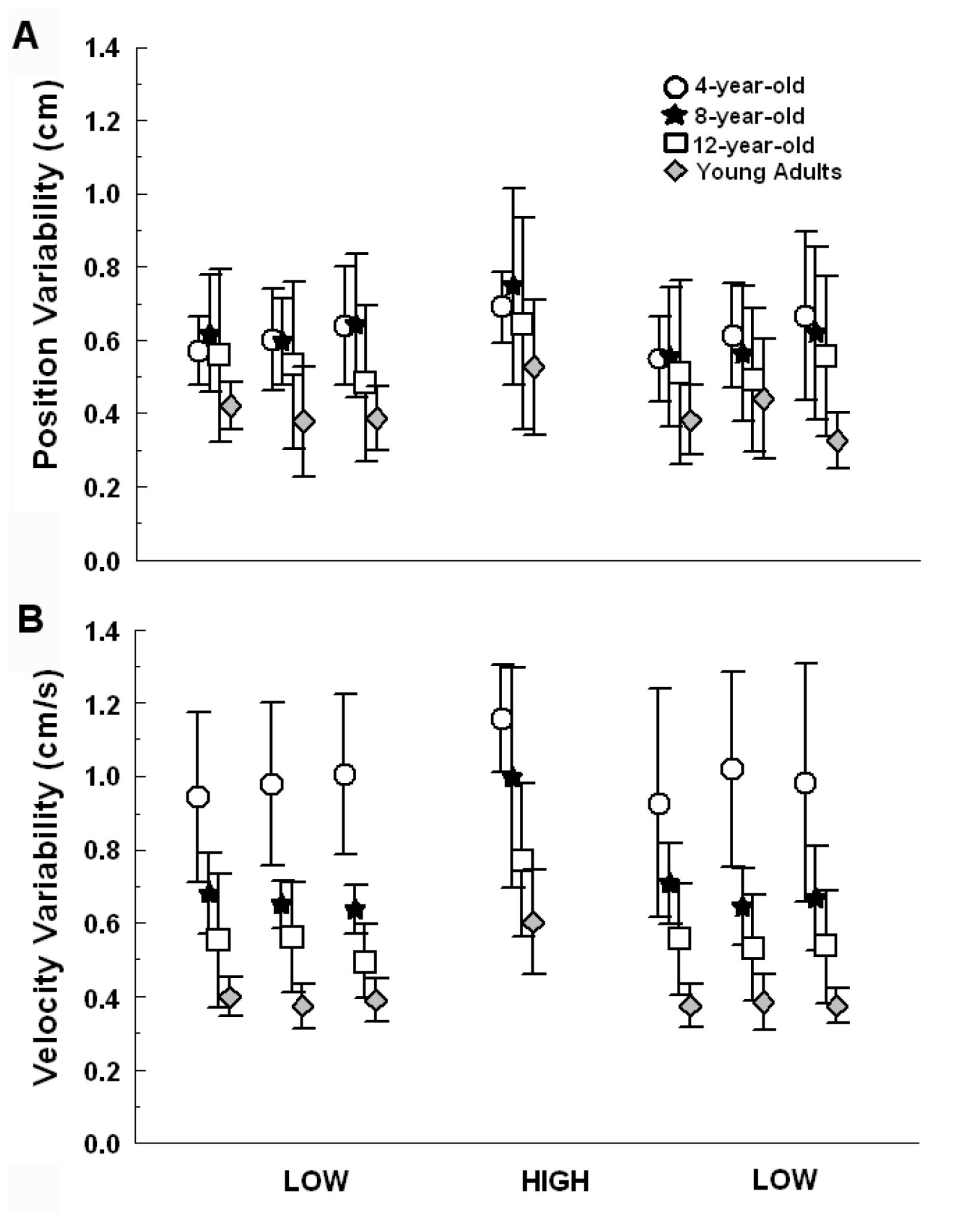
Position and velocity variability. Position (A) and velocity variability values (B) for each age group (4-, 8- and 12-year-old and young adults) across all trials. Values are mean ± SD.

Figure 3 depicts position (Figure 3a) and velocity variability (Figure 3b) for each age group across all trials. Sway variability at frequencies other than the stimulus frequency (residual variability) decreased with age and was largest in the high amplitude/velocity trial. ANOVA showed position variability trial, F(6,216) = 5.104, p < 0.001, and group effect, F(3,36) = 7.934, p > 0.001, but no group and trial interaction, F(18,216) = 0.907, p > 0.05. Post-hoc tests indicated larger sway variability for the 4- and 8-year-old groups when compared to young adults (p < 0.001), and larger sway variability for all groups during the high amplitude/velocity trial condition when compared to low amplitude/velocity trials (p < 0.05). 

An ANOVA of the velocity variability also revealed group, F(3,36) = 44.57, p < 0.001, and trial effect, F(6,216) = 31.335, p < 0.001, but no group and trial interaction, F(18,216) = 1.419, p > 0.05. Sway velocity variability of the 4-year-old group was increased when compared to the 8-year-old (p < 0.001), 12-year-old (p < 0.0001), and young adult groups (p < 0.0001). Moreover, the 8-year-old group demonstrated increased sway velocity variability when compared to the 12-year-old (p < 0.03) and young adult groups (p < 0.002). Young adults presented the lowest sway velocity variability when compared to all the children’s groups. Additionally, body sway of all groups showed larger velocity variability during the high amplitude/velocity trial than during all other trials (p < 0.0001).

## Discussion

 The present study investigated how children’s postural control adapts to changes in the surrounding visual environment and whether they use previous experience to respond to following environmental changes. Overall our results indicated that children as young as 4 year olds are capable of quickly decreasing body responses due to amplitude/velocity changes in visual stimulus, indicating sensorimotor re-weighting capability. Despite showing re-weighting of multiple sensory cues, younger children, 4 and 8 year olds, do not reduce sensory stimulus manipulation influence at the same magnitude as 12-year-old children and young adults. Moreover, being unable to avoid as much sensory influences as older children, younger children are more influenced by visual stimuli and, therefore, display increased postural sway responses when compared to older children. Finally, 4-year-old children do not use previous experience to adapt their following postural responses as older children and young adults do.

Postural development signature is the reduction of the magnitude of body sway that occurs with age [5,10,11,15,36-38]. It has been suggested [3] and recently shown [5,32] that developmental changes in postural control functioning is related to the acquisition and refinement of a flexible and adaptable relationship between sensory information and body sway. Our results not only corroborate this suggestion but also show developmental changes in a possible mechanism underlying the adaptive postural control; that is, the multisensory re-weighting process is involved in properly increasing/decreasing the importance and use of the available sensory cues to furnish reliable sensory information as environmental conditions change [26].

Based upon these results, it is suggested that the CNS of 4- and 8-year-old children is already able to identify the visual amplitude/velocity change; decrease postural response to the visual stimulus (reduced visual gain) and hypothetically increase the weight of other sensory inputs (i.e. vestibular and somatosensory). This hypothetical sensory switch is interpreted as sensorimotor re-weighting [26] and the theoretical framework is based upon the stimulus gain changes. This hypothetical system could explain how the CNS down-weights the influences of inaccurate sensory cues that might threaten postural control [25]. Clearly, such a mechanism is already functional in 4-year-old children who adaptively responded to changing sensory environments, down-weighting visual influences when the visual cue was increased in terms of magnitude and velocity. Such a mechanism was also recently observed caused by continuously changing visual cues [32] and also caused by both visual and somatosensory cues manipulation in the postural response of 4-year-old children [17]. These recent results challenge previous suggestions that sensorimotor integration in children takes place only later in life [7,14] and, conversely, indicate that sensorimotor integration and adaptive behavior to multisensory cues manipulation is performed by children as young as 4 years old.

Despite showing re-weighting of multiple sensory cues, younger children do not exhibit re-weighting responses with the same magnitude as older children. Four- and eight-year-old children display reduced visual stimulus influence due to amplitude and velocity increases (lower gain values) but not at the same magnitude as 12 year olds and adults. Therefore, skillful responses to stimulus changes in sensory stimuli seem to be age-related with 4- and 8-year-old children not showing fully developed responses as observed in 12-year-old children. Such differences in the magnitude of the re-weighting process might explain why infants [6,39] and children [7,40] might have during postural orientation and equilibrium compromised when they are exposed to sensory conflicts. It seems that the CNS of infants and young children, when exposed to large or complex conflicting stimulus manipulations, is incapable of accurately uncoupling incongruent sensory stimuli, as previously suggested by Barela et al. (2003) and, thereby, disrupts or compromises postural stability.

Our results suggest that adaptive sensorimotor coupling is related to sway magnitude performance. In this study, as in previous studies [5,36] 12-year-old children displayed a sway magnitude similar to that of young adults. In addition, gain values were similar between 12-year-old children and young adults in all conditions in which the room was oscillating, suggesting similar adaptive behavior in both groups. Thus, as suggested in previous studies [3,5,16], and clearly demonstrated by the present results, adult-like sway magnitude observed at 12 years of age is related to a flexible postural control which adaptively re-weighs sensory information to the changing cues available in the environment. This, furthermore, might be considered an underlying developmental mechanism of the CNS that leads to postural control development.

Interestingly, we have examined that body sway magnitude, observed in the stationary room trial, is related to sway magnitude in the moving room trials. We have employed an ANCOVA, having mean sway amplitude in the stationary trial as covariate, and no differences among groups and group and condition interaction were observed in the mean sway magnitude in the trial that the room was moved. Therefore, larger sway in maintaining upright stance in normal (stationary) visual condition is associated with larger sway for those who display larger sway in the visual manipulation condition. Such relation between the use of the most useful sensory available cues and body sway magnitude has been previously observed [5] and adult-like behavior also reached around the age of 12-year of age. In the stationary visual condition young children cannot extract and use the most relevant sensory cues in order to achieve a precise body dynamics framework. Similarly, in the moving room trials, in which sensory cues provide conflicting information about body dynamics, changing from one sensory cue source to others is more critical, larger body sway is still observed for younger children.

In spite of the fact that children’s postural sway magnitude and adaptive behavior are similar to young adults, 12-year-old children still display different temporal relationships between their postural response and a moving room when compared to young adults and larger velocity sway variability. Interesting results were also found regarding the temporal relationship between visual information and body sway. Our results demonstrated that body sway of 4-year-old children were ahead of the visual stimulus while 8- and 12-year-old children maintained body sway close to 0.2 Hz (the driving frequency), which was also different when compared to young adults who swayed behind the moving room. Furthermore, phase values tended to be increased for all age groups when the stimulus amplitude was increased. This amplitude dependence of the temporal stability is not consistent with the predictions about phase from the adaptive postural model [26,27] which suggests that phase remains roughly constant when stimulus amplitudes are changed. Previous results have shown this phase pattern for adults and elderly individuals [20,41] which has been suggested to be caused by an increase in the stiffness of the postural control [20]. However, these aspects remain unknown and need to be examined further. Similarly, the reasons why children lead visual stimuli needs further examination, which might indicate different temporal functioning and adaptive behavior in children.

Our results also demonstrated that sway variability increased, even in adults (velocity sway variability), with increasing stimulus amplitude/velocity (Figure 3). This finding indicates that the CNS cannot fully ignore the available sensory information and, in order to minimize sensory information influences, body sway is produced in frequencies other than the driving stimulus frequency. Moreover, sway variability is increased in children, as previously observed [3,5], but decreases with age, approaching adult-like levels near the age of 12 years old (at least with respect to the position sway variability component). Increased sway variability in children’s postural control was suggested to occur because children are incapable of uncoupling unreliable sensory information available in the environment [3,5]. Therefore, sway variability would reflect the children’s capabilities to adaptively re-weight sensory cues by coupling/uncoupling reliable/unreliable information in order to skillfully maintain upright stance during changing environmental sensory cues.

Adaptive multisensory re-weighting is crucial in performing a task at hand but also such an adaptation experience needs to be carried over for upcoming conditions. It was speculated that it is functionally advantageous for the CNS to decrease the coupling between body sway and altered visual stimuli to avoid postural instability in the case of another stimulus amplitude change [26], that is, carrying over past experiences to other situations. Interestingly, our results indicate that younger children (4- and to some degree 8-year-olds) do not benefit from previous experiences to adapt to new amplitude/velocity stimulus changes. In particular, after the second stimulus transition, high-to-low amplitude/velocity change – fourth and fifth trials, 12-year-old children and young adults never return to gain values observed in the first three trials. Conversely, 4-year-old children showed comparable gain values in the fifth trial and 8-year-old children in the sixth trial. Therefore, younger children not only exhibit less calibrated adaptive sensory down-weighting but also do not carry such adaptive experience to subsequent adaptations when exposed to similar environmental conditions.

A few studies have indirectly indicated flexible coupling between sensory information and body sway in infants [2,42-45] and in children [5,31,40] when they are repeatedly exposed to altered sensory conditions. In doing so, infants and children show adaptive sensorimotor re-weighting, as shown in this study, but indeed such adaptation requires repeated exposure to the changing environmental conditions. Also, infants and younger children seem to adjust their responses to continuously changing environmental conditions based upon past experiences [3], requiring longer exposure time to build up an internal representation of such adjustments. In other words, experiences are crucial in order to identify and select useful available sensory information, from the many and multiple sensory cues available in the environment, requiring stable but at the same time flexible coupling between sensory information and motor activity. Stable and flexible coupling require adaptive and dynamic multiple sensorimotor re-weighting that is subsequently acquired later in life and built upon the experiences that infants and young children resolve in their daily activities.

In summary, this study indicated that children as young as four years of age have developed the adaptive capability to quickly down-weight visual information. However, the higher gain values and residual variability observed in 4- and 8-year-old children suggest that these children have not fully calibrated their responses to the levels that are observed for older children, at least, until the first decade of life. Moreover, younger children do not carry over previous experiences from the sensory amplitude to the upcoming conditions as 12 year olds and adults do. Therefore, our results clearly indicate age-related changes in the adaptation of the postural control system to the continuously changing surrounding environment. Such adaptive behavior may be crucial for the development of a stable upright stance that enables the acquisition of flexible postural control. 
